# Daily blood pressure profile and blood–brain barrier permeability in patients with cerebral small vessel disease

**DOI:** 10.1038/s41598-022-11172-1

**Published:** 2022-05-11

**Authors:** L. A. Dobrynina, K. V. Shamtieva, E. I. Kremneva, M. R. Zabitova, B. M. Akhmetzyanov, E. V. Gnedovskaya, M. V. Krotenkova

**Affiliations:** 1 3rd Neurology Department with Psychological and Speech Therapy Group – cerebrovascular diseases and cognitive impairments, Research Center of Neurology, Moscow, Russia; 2 Department of Radiology, Research Center of Neurology, Moscow, Russia

**Keywords:** Cerebrovascular disorders, Preventive medicine, Magnetic resonance imaging

## Abstract

Cerebral small vessel disease (CSVD) plays an important role in cognitive impairment, stroke, disability, and death. Hypertension is the main risk factor for CSVD. The use of antihypertensive therapy has not resulted in the expected decrease in CSVD complications, which may be related to the underestimation of significance of daily blood pressure profile for blood–brain barrier (BBB) permeability. 53 patients with CSVD of varying severity (mean age 60.08 ± 6.8 years, 69.8% women, subjects with treated long-standing hypertension vs. normotensive subjects − 84.8% vs. 15.2%) and 17 healthy volunteers underwent ambulatory blood pressure monitoring (ABPM) and MRI, including T1-weighted dynamic contrast-enhanced magnetic resonance imaging for assessing BBB permeability. Most of ABPM parameters in CSVD patients did not differ from controls, but were associated with the severity of white matter hyperintensity (WMH) and the total CSVD score. BBB permeability in normal-appearing white matter (NAWM) and grey matter (GM) was significantly higher in CSVD patients, and the severity of BBB permeability remained similar in patients with different stages of WMH. Among BBB permeability parameters, the area under the curve, corresponding to an increase in the contrast transit time in NAWM, had the greatest number of correlations with deviations of ABPM parameters. BBB permeability in CSVD is a universal mechanism of NAWM and GM damage associated with a slight increase in ABPM parameters. It is obvious that the treatment of hypertension in patients with not severe WMH should be more aggressive and carried out under the control of ABPM.

## Introduction

Cerebral small vessel disease (CSVD), associated with age and vascular risk factors, is the main cause of vascular cognitive impairment, mixed neurodegenerative and vascular dementia, as well as a significant cause of stroke, disability and mortality in adult population^1–3^.

Hypertension is the main risk factor for age-related CSVD^4–6^. In most cases, the severity of hypertension correlates with MRI markers of CSVD, such as white matter hyperintensity (WMH) and lacunae^4,7^. The leading mechanism of brain damage in patients with chronic hypertension is hypoperfusion secondary to arteriolosclerosis, which is characterized by the loss of smooth muscle cells, accumulation of fibrotic hyaline deposits, thickening of the blood vessel walls, and luminal narrowing^8,9^.

Modern antihypertensive therapy, aimed primarily at preventing remodelling of small arteries and, accordingly, increasing blood flow, has led to a decrease in the incidence of stroke^10,11^, but not the prevalence of cognitive impairment^12,13^. This can partly be explained by a lack of or a faint effect of antihypertensive therapy on the increased blood–brain barrier (BBB) permeability^14^, which, along with ischemia, is another significant mechanism of brain damage in hypertension^15–17^. BBB damage in patients with hypertension is considered to be the main mechanism for the initiation and progression of CSVD and the development of forms mixed with neurodegeneration^15–17^. In these cases, hypertension is both a factor in BBB damage^18^ and a consequence of the damage to the cerebral autonomic centres caused by the high BBB permeability^19^.

For a long time, BBB damage with high permeability has been considered mainly as a failure of cerebral autoregulation due to high blood pressure in acute and chronic hypertension^20,21^. Further experiments on the spontaneously hypertensive rats and stroke‐prone spontaneously hypertensive rats have proven that the mechanism of BBB damage in CSVD is universal and can be observed in milder hypertension stages^22^. One of the possible explanations may be the effect of blood pressure variability on the high BBB permeability. This assumption is consistent with recent studies that have noted the importance of blood pressure variability in the development of CSVD, including in controlled hypertension according to outpatient measurements^23,24^.

In vivo study of the role of this mechanism in CSVD development has become possible with T1-weighted dynamic contrast-enhanced magnetic resonance imaging (DCE-MRI), enabling a quantitative assessment of BBB permeability^25,26^. Increased BBB permeability was found in normal-appearing white matter (NAWM) as compared to controls^27,28^; in WMH and adjacent NAWM, correlating with the severity of WMH, hypertension, age and leading to the delayed reduction in cognitive capacity^25^.

Thus, hypertension and increased BBB permeability are of great importance in the development of CSVD^17,25,27,28^, but antihypertensive therapy has insufficient effect on reduction of cognitive impairment^12,13^. Of particular interest is the clarification of the relationship between the features of hypertension, which is well controlled by outpatient measurements, and BBB permeability in patients with CSVD and cognitive impairment. The study aims to evaluate the relation between daily blood pressure profile and BBB permeability in patients with CSVD and cognitive impairment.

## Materials and methods

### Participants and ethics

The study included patients aged 46–70 years with cognitive and other neurological complaints, such as gait and balance problems, mood disorders or residual symptoms after stroke, brain changes on MRI corresponded to CSVD (WMH, lacunae, enlarged perivascular spaces, microbleeds and cerebral atrophy)^29^. Patients with low WMH burden (Fazekas scale score 1) were included in the study if they had hypertension stage 2 or 3 and/or ≥ 1 lacuna.

Exclusion criteria: (1) cognitive impairment due to probable Alzheimer's disease according to the U.S. National Institute on Aging criteria^30,31^; (2) patients with small subcortical infarcts/lacunes < 3 months after an acute cerebrovascular event; (3) CSVD due to other independent causes (genetic, inflammatory, thrombophilic, systemic, toxic, history of severe migraines); (4) a different cause of stroke and concomitant brain pathology other than CSVD; (5) > 50% atherosclerotic stenosis of the extra- or intracranial arteries; (6) serious medical condition—cardiac (ejection fraction < 50%), endocrine (diabetes mellitus type 1 or 2 with severe vascular complications, uncompensated thyroid disorder), renal (chronic kidney disease with glomerular filtration rate < 30 ml/min), etc.; (7) contraindications for MRI.

The control group consisted of volunteers with no clinical or MRI evidence of vascular and degenerative brain pathology, no hypertension in the medical history and during Ambulatory Blood Pressure Monitoring (ABPM), and matched for age and gender. Controls with hypertension according to ABPM were excluded from the study, in accordance with the European Society of Hypertension recommendations: awake blood pressure was ≥ 135/85 mmHg and/or asleep blood pressure was ≥ 120/70 mmHg, or if blood pressure increased by more than 24% over time during exertion^32^.

In total 53 patients (37 women, average age 60.1 ± 6.8 years) and 17 healthy volunteers (12 women, average age 56.7 ± 6.7 years) were enrolled in the study.

Traditional vascular risk factors, such as hypertension^33^, hypercholesterolemia, obesity, diabetes mellitus and smoking were assessed in the patients and controls.

The study was approved by the Local Ethics Committee of the Research Centre of Neurology № №2–4/16 dated 17.02.2016 and performed in accordance with the principles of the Declaration of Helsinki. All subjects signed an informed consent form for participation in the study.

### Ambulatory blood pressure assessment

All participants underwent ABPM with an automated device (LLC DMS Advanced Technologies*,* Moscow) based on oscillometric method. Patients underwent ABPM during hospitalization with blood pressure measurement every 30 min during the day (8:00 am to 10:00 pm) and every 60 min during the night (10:00 pm to 8:00 am). The ABPM device inflatable cuff was placed on the non-dominant upper limb. In all cases, at least 70% of the measurements were suitable for analysis. We calculated mean 24-h systolic blood pressure (SBP) and diastolic blood pressure (DBP); mean, standard deviation and maximal values of awake and asleep SBP and DBP; and blood pressure load parameters as the percentage of readings in a given period (24-h, day, or night), which exceeded the normal levels for awake and asleep SBP and DBP^32^.

The grade of hypertension was determined from the medical history and was adjusted according to ABPM results. During hospitalization patients continued their antihypertensive therapy.

### Neuroimaging

Imaging was carried out in a Siemens MAGNETOM Verio 3 T scanner (Siemens Medical Systems, Erlangen, Germany) with a standard 12-channel matrix head coil. To evaluate STRIVE criteria^29^, patients and the control group underwent axial spin echo T2-weighed imaging (TR 4000 ms; TE 118 ms; slice thickness 5.0 mm; duration: 2 min 02 s); sagittal 3D T2 FLAIR (TR 6000 ms; TE 395 ms; 1.0 mm^3^ cubic voxel; duration: 7 min 12 s); sagittal 3D T1-weighed imaging (TR 1900 ms; TE 2,5 ms; 1.0 mm^3^ cubic voxel; duration: 4 min 16 s); diffusion MRI (DWI) using axial spin-echo echo-planar imaging sequence with two b-values—0, 1000 s/mm^2^ (TR − 4000 ms, TE − 100 ms, slice thickness − 4 mm, duration: − 1 min 20 s); axial susceptibility weighted imaging sequence (SWI) with magnitude and phase images reconstruction (TR 28 ms; TE 20 ms; slice thickness 1.2 mm; duration: 8 min 12 s).

Two neuroradiologists evaluated MR images in a standardized manner, blinded to clinical information. No STRIVE criteria were found in volunteers from control group. There were no acute or recent small lacunar infarcts based on DWI analysis in patients with CSVD. MRI presence of lacunes, white matter hyperintensities, microbleeds, and perivascular spaces were summed in a score of 0–4 representing all CSVD features combined^34,35^.

The Fazekas Scale^36^ was used to quantify T2 FLAIR white matter hyperintensities (WMH) (score 0–3) as well as semi-automatic WMH segmentation using LST toolbox (http://www.applied-statistics.de/lst.htm) for SPM12 (http://www.fil.ion.ucl.ac.uk/spm) with further manual correction using ITK-SNAP viewer (http://itksnap.org). The obtained data were saved as a binary mask, which was taken into consideration when the NAWM mask was subsequently created to calculate BBB permeability.

DCE-MRI was performed for BBB leak assessment. After two T1-weighted volumetric interpolated breath-hold examination (T1-VIBE) acquisitions (flip angles 2 and 15) for pre-contrast T1-mapping, we injected gadodiamide (Omniscan; GE Healthcare) 0.2 mL/kg (i.e., 0.1 mmol/kg body weight) at a rate of 3 mL/s intravenously via injection pump and then performed continuous serial acquisitions of 100 volumes of T1-VIBE images for 15 min 33 s. The scanning parameters were: TR − 8.6 ms, TE − 4 ms, field of view − 250 mm, matrix − 256 × 230 px, flip angle − 15°, and slice thickness − 3.6 mm.

### Image processing

**Figure 1:**
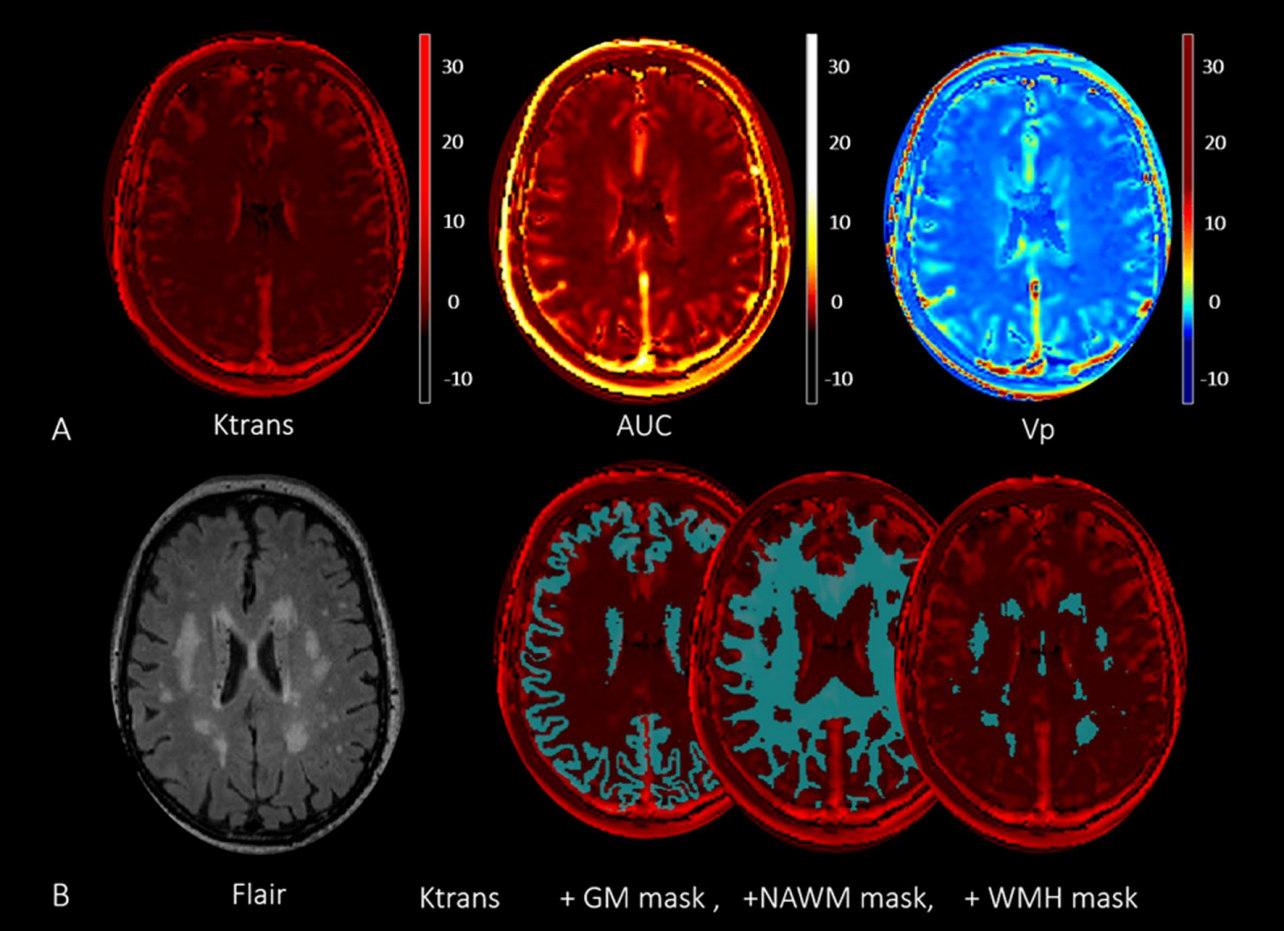
Example of MRI data of CSVD patient. Ktrans, AUC and Vp maps (**A**). T2**-**Flair image and superimposion of GM, NAWM and WMH masks over the individual ktrans permeability map (**B**).

The entire DCE-MRI dataset underwent preliminary processing using the NordicNeuroLab software (NordicICE, Norway). This included automatic correction of motion artefacts, correction of pre- and post-contrast data in the dynamic series, concentration of contrast agent in the brain tissue calculation using relative signal change and T1 mapping. Individual vascular input functions were derived semi-automatically from the superior sagittal sinus^37^. The hematocrit, contrast agent dose and relaxivity of the contrast agent were set individually for each patient. The Patlak pharmacokinetic model was used to assess the low BBB permeability in CSVD resulting in Ktrans (volume transfer coefficient), Vp (fractional blood plasma volume) maps, and AUC (area under the curve—corresponding to increased contrast transit time in the brain) maps (Fig. 1A).

Once permeability parameter maps were obtained, further data processing was performed in SPM12 (http://www.fil.ion.ucl.ac.uk/spm). This included the following steps: coregistration of each subject’s permeability parameter maps and the T1-weighted images; segmenting the T1-weighted images into grey matter and white matter, followed by the correction of obtained images using WMH masks based on a MatLab script (https://matlab.ru/), resulting in the binary images of the corrected grey and white matter. Permeability parameters were calculated in ITK-SNAP (http://itksnap.org) separately for grey matter (GM), NAWM and WMH by superimposing the relevant masks over the individual permeability maps (Fig. 1B). BBB permeability parameters have very low values (10^−4^), which do not visually differ, therefore, an analysis of the data of a patient with CSVD stage 2 according to Fazekas Scale is given as an example.

### Statistical analysis

Statistical analysis was performed using IBM SPSS 23.0 (IBM SPSS Statistics, version 23.0, IBM Corp., Armonk, NY, USA) and R 3.4.3 (R Foundation for Statistical Computing, Vienna, Austria) software. Data are presented as n (%) for categorical variables or as mean ± standard deviation (SD) or median [interquartile range (IQR)] for quantitative data. Differences between groups were determined using χ2, independent samples t-test or Mann–Whitney test, univariate analysis of variance or Kruskal–Wallis test with Bonferroni correction, where appropriate. In all cases, two-way statistical criteria were used. The null hypothesis was rejected if *p* < 0.05. Pearson's correlation coefficient and Spearman's correlation were used to assess the relationship between parameters.

## Results

CSVD and the control groups were matched for age and gender, and consisted predominantly of women (Table 1). Vascular risk factors were comparable except for hypertension, which was the dropout criterion for the control group.

**Table 1:** Main demographic parameters and risk factors in patients with CSVD and controls.

Parameters	CSVD (n = 53)	Control group (n = 17)	*p*
Gender, women (n, %)	37 (69.8%)	12 (70.5%)	0.951
Age, years (mean ± SD)	60.08 ± 6.8	56.71 ± 6.7	0.079
Hypertension (n, %)	45 (84.8%)		
Grade of Hypertension (n, %) Grade 1 Grade 2 Grade 3	12 (22.6%) 12 (22.6%) 21 (39.6%)	– – –	
Antihypertensive therapy (n, %) Irregular use 1 drug 2 drugs 3 drugs ≥ 4 drugs	4 (8.9%) 11 (24.4%) 20 (44.4%) 9 (20%) 1 (2.3%)	– – – – –	
Diabetes mellitus type 2 (n, %)	10 (18.9%)	0 (0%)	0.053
Hypercholesterolemia (total cholesterol > 6, 2 mmol/L or statin use) (n, %)	31 (58.5%)	8 (47%)	0.345
Obesity (body mass index > 30 kg/m^2^) (n, %)	22 (41.5%)	5 (29.4%)	0.373
Smoking (n, %)	13 (24.5%)	7 (41.2%)	0.186

Most of the patients in the main group (84.8%) had hypertension of varying severity and were taking one or more antihypertensive drugs.

The main disease symptoms were cognitive impairment, gait disturbances unrelated to post-stroke hemiparesis, and MRI changes including WMH, lacunae, microbleeds and dilated perivascular spaces (Table 2).

**Table 2:** Clinical symptoms and MRI signs in patients with CSVD.

Parameters	CSVD (n = 53)
Cognitive impairment (n, %): Subjective Mild Dementia	53 (100%) 22 (41.5%) 24 (45.3%) 7 (13.2%)
Gait disturbances, unrelated to hemiparesis (n, %):	29 (54.7%)
Urinary disorders (n, %)	21 (39.6%)
History of stroke (n, %):	25 (37.9%)
WMH, Fazekas Score (n, %) Score 1 Score 2 Score 3	16 (30.2%) 16 (30.2%) 21 (39.6%)
Lacunae (n, %)	26 (49.1%)
Microbleeds (n, %)	25 (47.2%)
Perivascular spaces (n, %)	53 (100%)
Total CSVD score (n, %) 1 sign 2 signs 3 signs 4 signs	0 (0%) 19 (35.8%) 25 (47.3%) 9 (16.9%)
Total WMH, cm^3^	13,830 [5747; 32145]

Differences in mean asleep DBP, and asleep SBP and DBP load were found when ABPM results were compared between subjects with CSVD and those in the control group (Table 3).

**Table 3:** ABPM results in subjects with CSVD and controls.

Parameters	CSVD (n = 53)	Control group (n = 17)	*p*
24-h SBP (mmHg)	120.1 [112.9; 127.5]	118.3 [110.1; 121.2]	0.160
24-h DBP (mmHg)	78.8 [73.3; 86.8]	75.9 [71.7; 76.6]	0.091
Mean awake SBP (mmHg)	122.8 [114.4; 132.4]	119.0 [111.7; 125.3]	0.386
Mean awake DBP (mmHg)	81.9 [76.3; 91.3]	77.4 [74.2; 78.3]	0.903
Maximal awake SBP (mmHg)	146 [137; 164]	142 [134; 148]	0.072
Maximal awake DBP (mmHg)	105.0 [94.0; 112.0]	99.0 [92.0; 106.0]	0.217
Awake SBP load (%)	4.6 [0.0; 20.8]	0.9[0.0; 3.9]	0.131
Awake DBP load (%)	13.6 [1.2; 54.6]	6.2 [0.8; 14.1]	0.075
Awake SD of SBP (mmHg)	10.1 [8.1; 13.8]	9.4 [7.9; 10.9]	0.381
Awake SD of DBP (mmHg)	9.3 [7.2; 11.5]	9.7 [7.4; 11.6]	0.903
Mean asleep SBP (mmHg)	114.0 [106.4; 120.9]	110.3 [102.7; 115.9]	0.169
Mean asleep DBP (mmHg)	72.9 [66.7; 80.0]	67.2 [64.4; 64.4]	**0.000**
Maximal asleep SBP (mmHg)	128.0 [124.0; 141.0]	125.0 [119.0; 136.0]	0.311
Maximal asleep DBP (mmHg)	87.0 [78.0; 96.0]	83.0 [76.0; 89.0]	0.103
Asleep SBP load (%)	17.3 [5.4; 45.0]	5.8 [0.0; 18.8]	**0.009**
Asleep DBP load (%)	57.2 [18.6; 94.4]	17.5 [13.8; 22.7]	**0.002**
Asleep SD of SBP (mmHg)	8.2 [5.9; 10.0]	8.4 [6.9; 10.8]	0.732
Asleep SD of DBP (mmHg)	8.1 [6.3; 10.0]	8.6 [7.2; 10.9]	0.304

Significant values are in bold.

ABPM results showed a good response to antihypertensive therapy in the main group. Most of ABPM parameters in the study (except for mean asleep DBP, asleep SBP load, and asleep DBP load) did not show intergroup differences corresponding to doctors’ and patients’ opinion that hypertension was well controlled as measured on the outpatient basis.

Although, there were found the relations of ABPM results with neuroimaging markers of CSVD (Table 4).

**Table 4:** Relationship between ABPM, WMH, and total CSVD score.

Parameters	Fazekas 1 (n = 16)	Fazekas 2 (n = 16)	Fazekas 3 (n = 21)	*p* for Fazekas score^a^	Correlation with WMH	Correlation with total CSVD score
24-h SBP (mmHg)	116.7 [112.0; 121.2]	114.5 [107.6; 120.9]	126.6 [124.2; 134.0]	0.040	0.317*	0.272*
24-h DBP (mmHg)	77.1 [73.0; 80.5]	76.1 [70.4; 81.7]	85.5 [80.0; 91.4]	0.031	0.287	0.290*
Mean awake SBP (mmHg)	119.5 [111.9; 126.5]	122.2 [114.4; 123.8]	129.7 [123.5; 137.3]	0.384	0.288	0.243*
Mean awake DBP (mmHg)	77.9 [75.9; 86.6]	80.0 [75.7; 84.9]	88.8 [81.3; 93.5]	0.591	0.292	0.339**
Maximal awake SBP (mmHg)	140 [135; 152]	145 [140; 151]	161 [144; 170]	0.038	0.332*	0.274*
Maximal awake DBP (mmHg)	96.5 [91.0; 107.5]	102.5 [93.0; 110.0]	111.0 [97.0; 118.0]	0.053	0.323*	0.176
Awake SBP load (%)	0.2 [0.0; 6.6]	1.0 [0.0; 7.8]	16.8 [5.4; 45.0]	0.110	0.185	0.284*
Awake DBP load (%)	31.8 [18.9; 64.9]	52.4 [6.2; 87.6]	94.0 [47.5; 100.0]	0.130	0.284	0.284*
Mean asleep SBP (mmHg)	109.0 [104.3; 115.6]	108.9 [98.9; 111.3]	119.3 [117.0; 130.7]	**0.000**	0.357*	0.255*
Mean asleep DBP (mmHg)	68.8 [65.0; 73.0]	70.4 [61.6; 79.4]	80.0 [72.7; 83.4]	**0.007**	0.364*	0.370**
Maximal asleep SBP (mmHg)	125.5 [123.0; 128.0]	124.0 [113.0; 135.5]	136.0 [129.0; 154.0]	**0.001**	0.087	0.161
Maximal asleep DBP (mmHg)	82.5 [76.0; 89.5]	82.5 [73.0; 96.0]	91.0 [87.0; 99.0]	0.054	–0.011	0.142
Asleep SBP load (%)	12.7 [5.6; 18.9]	5.5 [0.0; 16.2]	45.0 [31.3; 98.3]	**0.000**	0.441**	0.387**
Asleep DBP load (%)	31.8 [18.9; 64.9]	52.4 [6.2; 87.6]	94.0 [47.5; 100.0]	0.019	0.338*	0.391**

^a^difference between groups were determined using univariate analysis of variance or Kruskal–Wallis test with Bonferroni correction, where appropriate.

*p < 0.05.

**p < 0.01.

Significant values are in bold.

Increased SBP and DBP values had significant relations with WMH load, based on Fazekas Scale score, and correlations with WMH volume and total CSVD score.

Relationships between the standard deviation of blood pressure, which correspond to the indices of variability, and neuroimaging markers of CSVD were not found either by comparative or correlation analysis, therefore these data are not included in the table.

**Figure 2:**
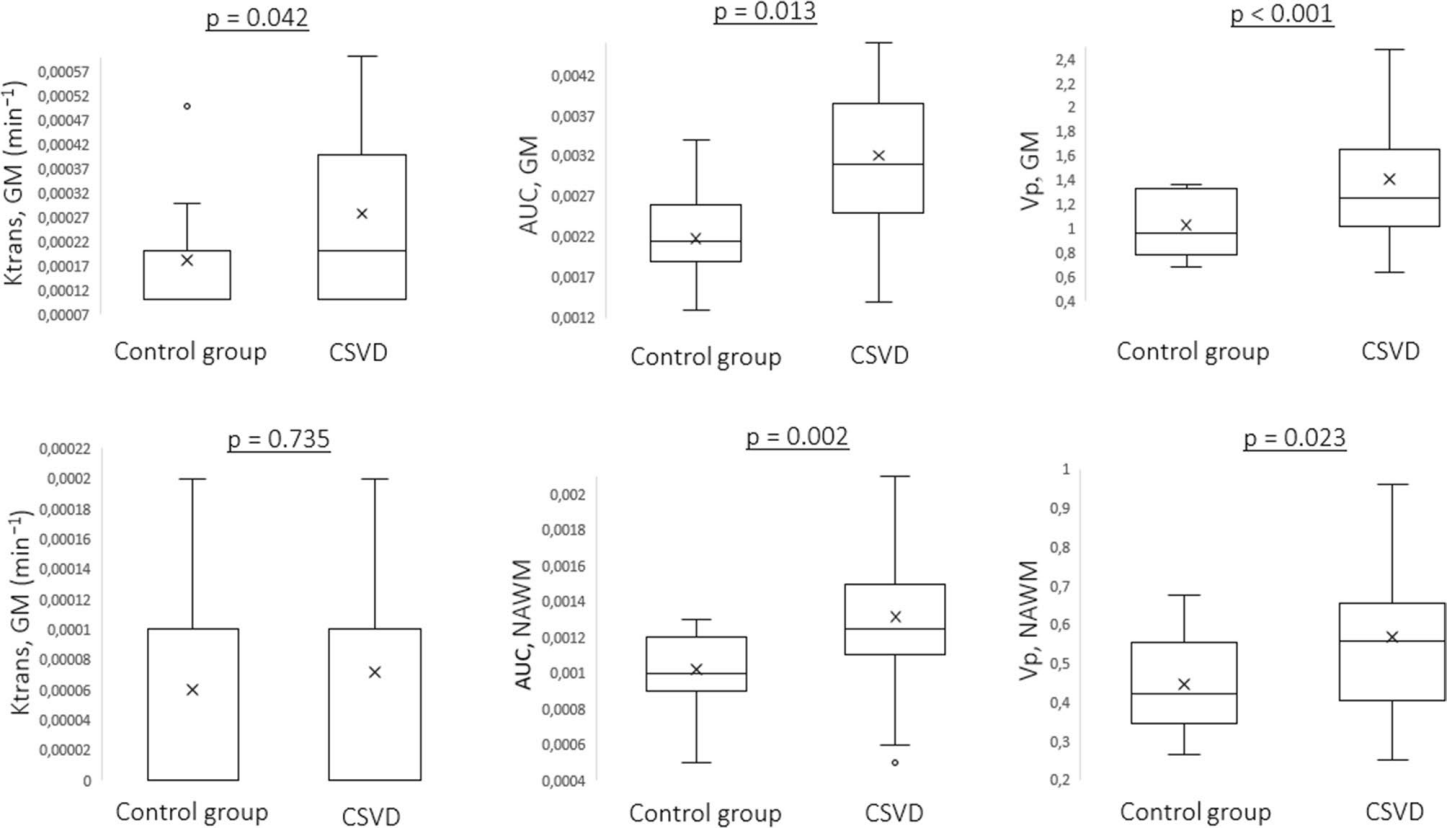
Differences in DCE-MRI parameters in patients with CSVD and controls.

To clarify the link between daily blood pressure fluctuations and BBB permeability, the last one was assessed using DCE-MRI in patients and controls (Fig. 2).

According to DCE-MRI, all the study parameters of BBB permeability in NAWM and GM, except for Ktrans in NAWM, were significantly higher in patients with CSVD than in controls.

BBB permeability decreased as WMH Fazekas score increased, with significant differences in Vp and AUC in WMH (Table 5).

**Table 5:** DCE-MRI parameters based on the Fazekas score.

Parameters	Fazekas 1 (n = 16)	Fazekas 2 (n = 16)	Fazekas 3 (n = 21)	*p*
Ktrans GM (min^−1^)	0.0003 [0.0002; 0.0004]	0.0003 [0.0001;0.0005]	0.0002 [0.0001;0.0004]	0.946
Vp GM	1.6954 [1.0997; 2.0808]	1.2464 [1.0814;1.6571]	1.1579 [1.0045;1.5523]	0.182
AUC GM	0.0035 [0.0025; 0.0044]	0.0033 [0.0028;0.0039]	0.0031 [0.0026;0.0033]	0.570
Ktrans NAWM (min^−1^)	0.0001 [0.0000; 0.0001]	0.0000 [0.0000;0.0001]	0.0001 [0.0000;0.0001]	0.361
Vp NAWM	0.7074 [0.4245; 0.7543]	0.5570 [0.4169;0.5999]	0.5212 [0.3893;06281]	0.420
AUC NAWM	0.0013 [0.0011; 0.0017]	0.0014 [0.0012;0.0015]	0.0012 [0.0011;0.0015]	0.940
Ktrans WMH (min^−1^)	0.0001 [0.0001; 0.0003]	0.0001 [0.0000;0.0002]	0.0001 [0.0000;0.0001]	0.563
Vp WMH	0.9323 [0.4574; 1.5027]	0.5266 [0.4054;0.6131]	0.4041 [0.2823;0.5034]	**0.003**
AUC WMH	0.0021 [0.0012; 0.0025]	0.0013 [0.0010;0.0017]	0.0010 [0.0008;0.0012]	**0.001**

Significant values are in bold.

Differences in the BBB permeability parameters in GM and NAWM depending on the WMH Fazekas score were not found.

Statistically significant correlations were seen between AUC in GM and NAWM, Vp in NAWM and the parameters of 24-h and awake SBP and DBP (Table 6).

**Table 6:** Correlation between ABPM results and DCE-MRI results (*p < 0.05, **p < 0.01).

Parameters	AUC, GM	Vp, NAWM	AUC, NAWM
24-h SBP	0.148	0.254*	0.321**
24-h DBP	0.201	0.226	0.283*
Mean awake DBP	0.251*	0.245*	0.331**
Maximal awake SBP	0.239*	0.331**	0.325**
Maximal awake DBP	0.265*	0.344**	0.336**
Awake SBP load (%)	0.284*	0.345**	0.459**
Mean asleep SBP	0.081	0.174	0.237*
Maximal asleep SBP	0.051	0.173	0.237*
Maximal asleep DBP	0.142	0.188	0.256*

Significant values are in bold.

Increased BBB permeability, as assessed by AUC, was correlated with mean awake DBP, maximal awake SBP and DBP, awake SBP load (%) in GM, as well as with mean awake DBP and asleep SBP, maximal awake and asleep SBP and DBP, awake SBP load (%) in the NAWM.

AUC, which characterizes the contrast delay in the brain, had the highest sensitivity out of BBB permeability parameters.

No significant correlations were found between ABPM results and BBB permeability parameters in WMH, so these data are not provided.

## Discussion

This study sought to clarify the relation between treated hypertension characteristics, assessed by ABPM, and BBB permeability, assessed by DCE-MRI, in patients with CSVD. Most of ABPM parameters in the study did not show intergroup differences, which confirms a good response to antihypertensive therapy in the main group. However, comparison of ABPM results with the severity of WMH based on Fazekas Score and its volume, as well as with total CSVD score, showed direct and significant relations. These data indicate the presence of certain mechanisms related to the abnormal ABPM parameters in patients compared with controls. Since a significant proportion of the studied patients had mild hypertension, as well as mild clinical and MRI signs of the disease, we could assume that BBB damage and its high permeability played a significant role in CSVD development. This hypothesis is based on the study results that indicate the special role of endothelial dysfunction with high BBB permeability as a mechanism of early CSVD^25,38–40^.

According to DCE-MRI, all the study parameters of BBB permeability in GM and NAWM, except for Ktrans in NAWM, were significantly higher in patients with CSVD compared to controls that is consistent with the previous studies^26–28,41^. There were no significant differences in BBB permeability in GM and NAWM between patients with WMH of varying severity. It should be noted that the results of previous studies regarding the significance of BBB permeability in the development of early or late CSVD are contradictory^25,28,41^. A reasonable explanation for our data may be that an increase in BBB permeability is possible with a relatively intact endothelium of small vessels in GM and NAWM in CSVD, corresponding the data about the universal mechanism of BBB damage in patients with CSVD and hypertension of varying severity^22^. On the other hand, the obtained data on a decrease in BBB permeability in the WMH may represent the conditions that characterize late-stage CSVD such as progressive endothelial death, impaired autoregulation due to small vessel remodelling, wall thickening and lumen narrowing, reduced microvascular perfusion^9,28^. This explanation is also supported by the fact that none of the abnormal ABPM parameters had relations with increased BBB permeability in the WMH. This result matches the different responses to elevated blood pressure in normotensive and hypertensive rats in following experiment. The hypertensive rats had higher permeability to sucrose which was absorbed more slowly by the brain, and the authors attributed this to changes in blood flow in hypertension^18,42^. AUC, which characterizes the contrast delay in the brain, had the highest sensitivity out of BBB permeability parameters. In our study, AUC in GM and NAWM had the most of relations with ABPM parameters. Although the latter in patients with CSVD did not differ from ones of the control group, we cannot exclude preceding rises in blood pressure that exceeds the upper threshold of cerebral autoregulation with an increase in BBB permeability^21^. It can be assumed that the use of antihypertensive therapy may change the upper threshold of cerebral autoregulation and the conditions for its disruption. This statement is indirectly supported by the fact that a greater reduction in blood pressure in elderly people with hypertension is associated with increased cerebral blood flow, corresponding to a shift in the autoregulation curve^43^. On the whole, these data support the necessity for more aggressive treatment of hypertension in patients with CSVD^44,45^. The risk of cardiovascular complications is decreased when blood pressure is reduced more aggressively, so the guidelines were rationally revised to the target SBP < 120 mmHg^46^. A recent randomized trial also supported this finding, as cerebral perfusion did not decrease in patients with severe CSVD when blood pressure was aggressively reduced, unlike in healthy controls^47^.

The obtained data on the universal nature of increased BBB permeability in the NAWM and GM in patients with CSVD indicate an ongoing pathological process in the small arteries, which leads to endothelial damage in relatively well-preserved small vessels. The connection between BBB permeability in NAWM and GM and elevated ABPM parameters indicated the importance of autoregulation dysfunction in promoting this mechanism. It is possible that the underestimation of the pathological mechanism of brain damage due to increased BBB permeability can partly explain the significance of hypertension in middle age for the development of cognitive impairment in the elderly^48,49^.

The lack of significant differences in ABPM results related to BBB permeability between patients and controls allow us to hypothesize the presence of additional factors of endothelial damage and increased BBB permeability alongside hypertension. These factors may be chronic inflammation^50^ or high salt sensitivity, which have been found to independently correlate with CSVD^51^.

The results of the study point out the necessity for more aggressive treatment of hypertension and repeat usage of ABPM as well as requirement for searching and affecting factors that potentiate the role of hypertension in CSVD development. It is obvious that further studies are needed on the effect of antihypertensive therapy on BBB permeability and its ability to protect the brain from damage in patients with CSVD.

## Data Availability

Raw data were generated at Research Center of Neurology. The data that support the findings of this study are available from the corresponding author upon reasonable request. Clinical, neurovisualization and statistical data will be available upon request from any qualified investigator.

## Abbreviations

CSVD: Cerebral small vessel disease
BBB: Blood–brain barrier
ABPM: Ambulatory blood pressure monitoring
SBP: Systolic blood pressure
DBP: Diastolic blood pressure
WMH: White matter hyperintensity
NAWM: Normal-appearing white matter
GM: Grey matter
MRI: Magnetic resonance imaging
DCE-MRI: T1-weighted dynamic contrast-enhanced magnetic resonance imaging
Ktrans: Volume transfer coefficient
Vp: Fractional blood plasma volume
AUC: Area under the curve
